# Bioactivity of essential oils: a review on their interaction with food components

**DOI:** 10.3389/fmicb.2015.00076

**Published:** 2015-02-09

**Authors:** Marianne Perricone, Ersilia Arace, Maria R. Corbo, Milena Sinigaglia, Antonio Bevilacqua

**Affiliations:** Department of the Sciences of Agriculture, Food and Environment, University of FoggiaFoggia, Italy

**Keywords:** essential oils, antibacterial, bioactivity, food composition, methods

## Abstract

Essential oils (EOs) are liquid preparations, produced from plant materials. Although EOs showed a promising bioactivity *in vitro*, they could interact in foods with some components (fats, proteins, carbohydrates) and pH, thus many authors have reported that a significant effect of EOs toward spoiling and pathogenic microorganisms could be achieved *in vivo* by using higher amounts of oils. Different methods can be used to assess the bioactivity of EOs (disk diffusion and agar or broth dilution methods); however, there is not a standardized test and researchers propose and use different protocols (evaluating the Minimal Inhibitory Concentration, studying the survival curves, analysis through the scanning electron microscopy, etc.). Thereafter, the scope of this review is a focus on interactions of EOs with proteins, carbohydrates, oils, NaCl, and pH, as well as a brief description on the different protocols to assess their bioactivity both under *in vivo* and *in vitro* conditions.

## INTRODUCTION

Synthetic antimicrobial agents and chemical food preservatives have been used since ancient times as an effective method for controlling food spoilage. Nowadays, consumer concerns toward chemical preservatives determine an increasing interest on some natural antimicrobials, like essential oils (EOs). EOs are liquid preparations produced from plant materials (flowers, buds, seeds, leaves, twigs, bark, herbs, wood, fruits, and roots) of temperate to warm countries, like Mediterranean and tropical areas. Only few of them are solid or resinous at room temperature; they are limpid, soluble in lipids or in organic solvents, with a generally lower density than that of water, with a pale yellow to emerald green or blue to dark brownish red color (Burt, 2004; Gutierrez et al., 2008).

These extracts were referred as EOs by Paracelsus von Hohenheim in 16th, who used the term “*Quinta essentia*” to design the active component of a drug and from the Latin *essentia* comes the term “essential” (Guenther, 1948).

EOs play a major role in plants and act as antibacterials, antivirals, antifungals, insecticides, and protect the plants from herbivores. It is possible to list ca. 3000 EOs, but only 300 of them are used in perfumes and make-up products (creams, soaps, etc.), sanitary products, dentistry, agriculture, as preservatives, and flavor additives for foods, as fragrances for household cleaning products and industrial solvents and as natural remedies (as mixtures with vegetal oil in massages or in baths, in aromatherapy, etc.; Burt, 2004).

Essential oils can be produced by expression, fermentation, enfleurage, or extraction, although hydro-distillation is the most common method (Speranza and Corbo, 2010). EOs and their active components possess antiviral, antimycotic, antitoxigenic, and insecticidal properties. **Table 1** reports the most important EOs, their aroma notes, and the target bacteria.

**Table 1:** Antimicrobial and aroma characteristics of essential oils (EOs; modified from Ayala-Zavala et al., 2009).

Essential oil	Major volatile constituents	Antimicrobial effect against	Aroma notes
Garlic root (*Allium sativum*)	Methyl disulfide, allyl sulfide, allyl disulfide, allyl trisulfide, trimethylene trisulfide, allyl tetrasulfide	*Bacillus cereus, Escherichia coli, Shigella* spp., *Vibrio parahaemolyticus, Yersinia enterolitica, Salmonella enterica* serovars Enteritidis, Infantis, Typhimurium, *B. subtilis, Enterococcus faecalis Alternaria alternata*	Pungent, spice

Cinnamon leaf (*Cinnamomum zeylanicum*)	Cinnamaldehyde, eugenol, copaene, *β*-caryophyllene	*E. coli, Pseudomonas aeruginosa, Ent. faecalis, Staphylococcus aureus, Staph. epidermidis,* methicillin-resistant *Staph. aureus, Klebsiella pneumoniae, Salmonella* sp., *Vibrio parahaemolyticus*	Sweet, wood, spice

Thyme (*Thymus vulgaris*)	Thymol, *p*-cymene, *γ* -terpinene, linalool	*B. cereus, Clostridium botulinum, Ent. faecalis, E. coli, Staph. aureus, Listeria monocytogenes*, *Aspergillus flavus, A. niger, K. pneumoniae, Ps. aeruginosa, Salmonella* sp.	Spice, citrus, wood

Oregano (*Origanum vulgare*)	Sabinyl monoterpenes, terpinen-4-ol, *γ*-terpinene, carvacrol, thymol	*B. cereus, B. subtilis, C. botulinum, Ent. faecalis, E. coli, Staph. aureus, A. niger, L. monocytogenes, K. pneumoniae, ps. aeruginosa, Salmonella* sp.	Spice, herb

Clove (*Syzygium aromaticum*)	Eugenol, eugenyl acetate, caryophyllene	*B. brevis, B. subtilis, Cl. botulinum, Ent. faecalis, Candida* spp., *A. flavus, A. niger, E. coli, K. pneumoniae, Ps. aeruginosa, Staph. aureus, Salmonella* spp., *L. monocytogenes*	Sweet, spice, wood

Basil (*Ocimum basilicum*)	Linalool, methylchalvicol, eugenol, methyl eugenol, methyl cinnamate, 1,8-cineole, caryophyllene	*B. brevis, E. coli, A. flavus, A. niger, Ent. faecalis, E. coli, K. pneumoniae, Ps. aeruginosa, Staph. aureus*	Fresh, sweet, herb, spice

Coriander (*Coriandrum sativum*)	2(E)-decanal, 2(E)dodecenal, linalool	*E. coli, L. monocytogenes, Lactobacillus plantarum, Staph. aureus*	Sweet, flower, spice, citrus

Citrus peel (*Citrus* sp.)	Limonene, linalool, citral	*A. niger, A. flavus, Penicillium verrucosum, P. chrysogenum*	Sweet, citrus

Laurel (*Laurus nobilis*)	1,8-cineole, α-terpinyl acetate, linalool, methyl eugenol	*Staph. aureus, B. cereus, Ent. faecalis*	Fresh, herb, spice

Ginger (*Zingiber officinale*)	β-sesquiphellandrene, zingiberene	*A. flavus, A. niger, Ent. faecalis, E. coli, K. pneumoniae, Ps. aeruginosa, Staph. aureus*	Pungent, spice

Rosemary (*Rosmarinus officinalis*)	Borneol, verbenone, camphor, a-pinene, 1,8-cineole	*A. flavus, A. niger, Ent faecalis, E. coli, K. pneumoniae, Ps. aeruginosa, Staph. aureus, L. monocytogenes, Lb. plantarum, Salmonella* spp., *B. cereus*	Fresh, herb, resinous

Peppermint (*Mentha piperita*)	Menthol, menthone, menthyl acetate, menthofurane	*B. brevis, Staph. aureus, Vibrio cholerae, Ent. faecalis, E. coli, K. pneumoniae, Ps. aeruginosa, A. flavus, A. niger*	Fresh, herb

Even though several studies were performed *in vitro* to assess antibacterial and antifungal properties of EOs, only few studies reported on their bioactivity *in vivo*; food components (fats, carbohydrates, proteins, salts) and pH could reduce the antimicrobial effects of EOs in food systems. In fact, the same effect observed *in vitro* is achieved in food matrix only with higher concentrations (Tyagi et al., 2014).

The scope of this review is to highlight the interactions of EOs with proteins, carbohydrates, NaCl, and pH as a preliminary step to optimize food applications; the last section deals with the different protocols to assess their bioactivity *in vivo* and *in vitro*.

## CHEMICAL COMPOSITION AND MECHANISM OF ACTION OF EOs

Essential oils are mixtures of 20–60 components at quite different concentrations, with some compounds at fairly high concentrations (20–70%), and others in trace amounts. The components at high concentrations (terpenes, terpenoids, molecules with an aromatic ring) play a major role in the antimicrobial/biological effect of EOs (Bakkali et al., 2008).

Some important compounds of EOs are mono and sesquiterpenes, carbohydrates, phenols, alcohols, ethers, aldehydes, and ketones (Speranza and Corbo, 2010). Phenolic compounds have also been recognized as bioactive components (Tabassum and Vidyasagar, 2013).

Essential oils with aldehydes or phenols as major components (cinnamaldehyde, citral, carvacrol, eugenol, or thymol) are the most effective, followed by EOs containing terpene alcohols (Bassolé and Juliani, 2012). EOs with ketones or esters (β-myrcene, α-thujone, or geranyl acetate) possess a lower activity (Dormans and Deans, 2000; Barros et al., 2009).

Although the major components of EOs are very important for their biological activity, minor components play a significant role, as they can strengthen the effects of major components, though antagonistic, and additive effects have also been observed (Bassolé and Juliani, 2012). **Table 2** reports some examples of combination of EOs toward a wide range of bacteria.

**Table 2:** Combination of components and EOs and their antimicrobial interactions against several microorganisms (modified from Bassolé and Juliani, 2012).

Pair	Organism	Methods	Interaction
Thymol/carvacrol	*Staph aureus*, *Ps. aeruginosa*	Half dilution	Additive
	*E. coli*	Checkerboard	Synergism
	*S. aureus*, *B. cereus*, *E. coli*	Checkerboard	Antagonism
	*Staph. aureus*, *Ps. aeruginosa*	Mixture	Additive
	*E. coli*	Checkerboard	Additive
	*Salmonella* Typhimurium	Mixture	Synergism
Thymol/eugenol	*E. coli*	Checkerboard	Synergism
Carvacrol/eugenol	*E. coli*	Checkerboard	Synergism
	*Staph. aureus*, *B. cereus*, *E. coli*	Checkerboard	Antagonism
Carvacrol/Cymene	*B. cereus*	Mixture	Synergism
Carvacrol/linalool	*L. monocytogenes*	Checkerboard	Synergism
Menthol/Geraniol Menthol/Thymol	*Staph. aureus*, *B. cereus*		Synergism
Cinnamaldehyde/ Carvacrol	*E. coli*	Checkerboard	Additive
	*Salmonella* Typhimurium	Mixture	Synergism
Cinnamaldehyde/ Thymol	*E. coli*	Checkerboard	Synergism
	*Salmonella* Typhimurium	Mixture	Synergism
Cinnamaldehyde/ Eugenol	*Staphylococcus* spp., *Micrococcus* spp., *Bacillus* spp., *Enterobacter* spp.	Mixture	Additive
*S. aromaticum/ R. officinalis*	*Staph. epidermidis*, *Staph. aureus*, *B. subtilis*, *E. coli*, *Proteus vulgaris*, *Ps. aeruginosa*	Mixture	Additive
*O. vulgare/O. basilicum*	*B. cereus*, *E. coli*, *Ps. aeruginosa*	Checkerboard	Additive
*O. vulgare/T. vulgaris*	*Ent. cloacae*, *Ps. fluorescens*, *L. innocua*	Checkerboard	Additive
*Cymbopogon citratus/ C. giganteus*	*E. coli*, *L. monocytogenes*, *Sh. dysenteriae*, *Staph. aureus, Salmonella* Typhimurium	Checkerboard	Synergism, additive

The composition of EOs relies upon the harvesting seasons and the geographical sources (Burt, 2004), as well as from the part of plant, e.g., EO from the seeds of coriander (*Coriandrum sativum* L.) shows a different composition from EO of cilantro, produced from immature leaves (Delaquis et al., 2002).

Essential Oils are lipophiles, thus they can easily enter cells, disrupt the membrane and/or permeabilize it. The most important signs of membrane permeabilization are the loss of ions and the reduction of potential, the collapse of proton pump and the depletion of ATP pool (Bakkali et al., 2008).

In eukaryotic cells, EOs cause depolarisation of mitochondrial membranes, influence Ca^2+^ channels and reduce pH gradient, affecting the proton pump and the ATP pool (Bakkali et al., 2008). The membrane becomes abnormally permeable resulting in leakage of radicals, cytochrome c, calcium ions, and proteins. Permeabilization of outer and inner mitochondrial membranes causes apoptosis and necrosis and finally cell death (Armstrong, 2006; Speranza and Corbo, 2010); in addition, EOs can cause the coagulation of cytoplasm and some damages to lipids and proteins (Burt, 2004).

Intrinsic and extrinsic conditions can be responsible of susceptibility and resistance of pathogens (Bajpai et al., 2012). It is not possible to propose a general hit for the susceptibility/resistance to EOs; however, Speranza and Corbo (2010) suggested some milestones:

• Gram-negative bacteria appear more resistant. This higher resistance could be attributed to the outer membrane.
• Lactic acid bacteria (LAB) are the most resistant Gram-positive bacteria. This resistance was attributed to ATP generation by substrate level phosphorylation.
• Among the Gram-negative bacteria, pseudomonads show high resistance to these antimicrobials.
• Essential oils are generally more active toward yeasts.

## ANTIBACTERIAL ACTIVITY OF EOs IN FOOD SYSTEMS

The bioactivity of EOs might be reduced by certain food components (fats, carbohydrates, proteins, water, salt, antioxidants, preservatives, other additives) and pH (Glass and Johnson, 2004; Gutierrez et al., 2008); moreover, some extrinsic factors (temperature, packaging in vacuum/gas/air, characteristics of microorganisms) play a crucial role (Skandamis and Nychas, 2000; Smith-Palmer et al., 2001). Different studies reported higher levels of bioactivity at acidic pHs, as at low pH EOs behave in a more hydrophobic way and enter more easily cells (Negi, 2012).

High concentrations of fats and/or proteins in foodstuffs may protect bacteria, as they could provide a protective layer and absorb EOs, thus decreasing their concentration and effectiveness in the aqueous phase; on the other hand, high water, and/or salt levels appear to facilitate the action of EOs (Smith-Palmer et al., 2001; Carson and Riley, 2003).

Gutierrez et al. (2008) studied the effect of food ingredients (potato starch-0, 1, 5, or 10%; beef extract-1.5, 3, 6, or 12%; sunflower oil-0, 1, 5, or 10%) and pH (4–7) on the antimicrobial efficacy of oregano and thyme. They focused on both the lag phase and the maximum specific growth rate of *L. monocytogenes*. Starch and sunflower oil exerted a negative effect on the biological activity of EOs, whilst proteins affected it in a positive way; finally, the highest activity was found at pH 5.

Cava et al. (2007) studied the antimicrobial activity of cinnamon and clove EOs against *L. monocytogenes* in milk and found that the biological activity was reduced by fat; these results are in agreement with the effects of EOs in full-fat and in low-fat soft cheeses (Smith-Palmer et al., 2001).

The effect of EOs could be reduced by increasing the amount of complex sugars (starch), whilst glucose and other simple sugars acted in a different way, thus EO application should be orientated to food products containing more simple sugars than complex carbohydrates (Gutierrez et al., 2008, 2009).

Another key factor for the biological activity of EOs is the physical structure of foods, which may limit and affect the antibacterial activity; e.g., *Salmonella* Typhimurium was inoculated in a broth and in a gelatine gel, both containing an EO. In the gel the effect of EO was reduced for its limited diffusion (Speranza and Corbo, 2010).

In many cases EO combinations showed additive effects, e.g., Gutierrez et al. (2008) combined oregano and basil or thyme toward *Escherichia coli* and *Pseudomonas aeruginosa,* with majoram toward *E. coli*, and majoram and thyme mixed with basil, rosemary or sage against *L. monocytogenes.* Moreover, Lambert et al. (2001) suggested that carvacrol and thymol acted as additive terms against *Staphylococcus aureus* and *P. aeruginosa.*

Some EOs, even at low concentrations, can have a negative impact on the sensory attributes, due to their low breakpoint for perception (Lv et al., 2011); therefore the need of higher concentrations in food is highly unfortunate and limits their application to spicy foods. An alternative approach is the use of EOs into active packaging, either encapsulated in polymers of edible and biodegradable coatings or entrapped in sachets able to slowly release the active compounds on food surface or in the headspace (Pelissari et al., 2009; Sánchez-González et al., 2011). Cerisuelo et al. (2014) tested some passive, active, and nanocomposite multilayer films; the performances of EVOH were low, as this matrix was not able to retain the active compounds. However, the inclusion of bentonite nanoparticles into EVOH active coatings increased the release rate and the retention ability.

In addition, another way to minimize the organoleptic effects of EOs is the preparation of nanoemulsions; this approach positively affects both the stability and the antimicrobial activity (Donsí et al., 2011).

Tyagi and Malik (2012) and Tyagi et al. (2012) proposed the use of EOs in the vapor phase, by combining bactericidal volatiles and ionizing sources. Since active compounds of EOs are highly volatile, the presence in gaseous form facilitates the solubilization of lipophilic monoterpenes in cell membranes.

Some papers focused on the combination of EOs with other treatments as reported by Tyagi et al. (2012, 2013); they tested lemon grass and mentha oils in combination with mild thermal treatment (55°C). Hence, this strategy significantly reduces oil dose requirement, offers a very useful synergy, as the increase of the temperature increases the amount of oil in the vapor phase, thus it enhances its antimicrobial activity.

## SOME CASE-STUDIES DEALING WITH THE APPLICATION OF EOs IN FOODS

### MEAT AND MEAT PRODUCTS

Eugenol and coriander, clove, oregano, and thyme oils were used to control pathogens and autochthonous spoilage flora in meat, as they caused a marked initial reduction in the viable cell number (Speranza and Corbo, 2010). As reported elsewhere fat reduced the bioactivity of EOs in meat products; in fact, some authors reported that thyme oil reduced significantly bacterial population of *L. monocytogenes* in zero and low-fat (90 g/Kg) beef hot-dogs, but not in full-fat hot-dogs (260 g/Kg; Lemay et al., 2002; Singh et al., 2004).

The new consumer preference toward hurdle technology suggests the potentiality of combining different elements to preserve foods; following this approach, Chouliara et al. (2007) combined oregano EO and modified atmosphere packaging (MAP) for the prolongation of the shelf life of fresh breast chicken meat, stored at 4°C. The effect of oregano EO (0.1 and 1% w/w) was evaluated in combination with two kinds of MAP [30:70 CO_2_:N_2_ (MAP_1_) and 70:30 CO_2_:N_2_ (MAP_2_)]. Samples treated with 1% oregano oil and packaged under both MAPs did not attain the critical level of cell count (7 log cfu/g) during a 25 day storage period.

### SEAFOOD PRODUCTS

As reported for meat, fat reduced the bioactivity of EOs in fish. Speranza and Corbo (2010) reported that the effect of oregano oil at 0.05 % (v/w) toward *Photobacterium phosphoreum* was stronger on cod filets than on salmon (a fatty fish). Some authors (Corbo et al., 2008; Del Nobile et al., 2009a) proposed combinations of EOs to improve the microbial stability of fish burgers. A mix containing 0.11% of thymol, 0.10% of grapefruit seed extract (GFSE) and 0.12% of lemon extract was proposed, as it increased the shelf life of fish burgers (stored under refrigeration and packaged in air) by 40%. Moreover the combined effect of the EOs and MAP was evaluated; samples were packaged in air and in three different gas mix compositions: 30:40:30 O_2_:CO_2_:N_2_, 50:50 O_2_:CO_2_, and 5:95 O_2_:CO_2_. The proposed packaging strategies inhibited the growth of mesophilic bacteria.

### VEGETABLES AND FRUITS

In vegetables the antimicrobial activity of EOs is enhanced by a decrease of storage temperature and pH (Smith-Palmer et al., 2001). The shelf life of unpasteurised fruit juices is limited by microbial enzymatic spoilage; moreover, these products could be contaminated by some pathogens. Some EOs could be used to prevent this kind of problem. Lemongrass and geraniol have been found effective against *E. coli*, *Salmonella* sp., and *Listeria* spp. in apple, pear, and melon juices (Raybaudi-Massilia et al., 2006).

Raybaudi-Massilia et al. (2009) used malic acid and EOs extracted from cinnamon, palmarosa, and lemongrass (0.3 and 0.7%) or their major compounds (eugenol, geraniol, and citral) to prolong the shelf life of fresh-cut “PieldeSapo” melon (*Cucumis melo* L.). EOs were entrapped into an alginate-based edible coating and used for a challenge test; *Salmonella* Enteritidis (10^8^ cfu/ml) was used as the target microorganism. This system was able to control the growth of the pathogen for at least 21 days (Ayala-Zavala et al., 2009). In addition, Rojas-Graü et al. (2007) entrapped lemongrass, oregano oil, and vanillin in an apple puree-alginate edible coating to prolong the shelf-life of fresh-cut Fuji apples. Vanillin (0.3%w/w) preserved sensory quality for at least 21 days at 4°C.

### DAIRY PRODUCTS

Orange, lemon, grapefruit, madrine, terpeneless lime, orange, D-limonene, terpineol, and geraniol were tested against *Salmonella* Senftenberg, *E. coli*, *S. aureus* and *Pseudomonas* spp. in different types of milk. Terpineol was the most effective oil *in vitro*, thus it was used in combination with orange oil for a validation in milk. The effect of terpineol oil was affected by fat content, showing a microbial reduction of 7 log cfu/ml in skimmed milk, 4 log cfu/ml in low butterfat milk and 3 log cfu/ml in whole milk (Fisher and Phillips, 2008).

Another approach was proposed by Bevilacqua et al. (2007) who studied the possibility of prolonging the shelf life of caprese salad using MAP (65:30:5 N_2_:CO_2_:O_2_) in combination with thymol. The combination of thymol dipping and MAP prolonged the shelf life by 8 days, without negative effects on the sensory quality and on the growth kinetics of LAB.

### CEREAL-BASED PRODUCTS

Natural active compounds were also applied to fresh pasta. Del Nobile et al. (2009b) used thymol, lemon extract, chitosan, and GFSE at different concentrations (2000 and 4000 ppm) to improve the microbiological stability of refrigerated amaranth-based fresh pasta. The oils were tested against mesophilic and psychrotrophic bacteria, total coliforms, *Staphylococcus* spp., yeasts, and molds. Chitosan and GFSE were the most promising compounds, whereas lemon extract was the less effective.

## METHODS TO ASSESS THE ANTIMICROBIAL ACTIVITY OF EOs

The methods to assess the antimicrobial activity of EOs could be grouped in three classes: diffusion, dilution, or auxographic methods (Rios et al., 1988). **Table 3** and **Table 4** report an overview of the most common protocols used to test the bioactivity of EOs. The most widely used test is NCCLS method, generally designed to test antibiotics but modified for testing EOs (Hammer et al., 1999; NCCLS, 2000); a filter disk is impregnated with the antimicrobial agent, placed on the surface of inoculated agar plates and an inhibition of growth is observed after incubation. This test is generally used for screening purposes, although its results rely upon many factors, like the method used to extract the EO from plant material, the volume of *inoculum*, the physiological phase of the microorganism, the kind of culture *medium*, pH, incubation time, and temperature. A modification of the method is the use of wells instead of a paper disk.

**Table 3:** Different methods used to test the antimicrobial activity of EOs (Burt, 2004).

Purpose	Test method
Screening for antibacterial activity	Disk diffusion (solid or vapor diffusion assay) Agar wells
Determination of the strength of antibacterial properties	Agar dilution method Broth dilution (visible growth, optical density/turbidity, absorbance, viable count, colorimetric and conductance/conductivity/impedance)
Determination of rapidity and duration of antimicrobial activity	Time-kill analysis/survival curves
Evaluation of the physical effects	Scanning electron microscopy

**Table 4:** Terms used in antibacterial activity testing reported in literature (from different literature sources).

Term	Definition presented in literature
Minimum inhibitory concentration (MIC)	Lowest concentration resulting in the maintenance or in the reduction of inoculum viability Lowest concentration required for the complete inhibition up to 48 h Lowest concentration inhibiting visible growth Lowest concentration resulting in a significant decrease in inoculum viability (>90%)
Minimum bactericidal concentration (MBC)	Concentration able to kill at least the 99.9% of the target. Lowest concentration at which no growth is observed after subculturing into fresh broth.
Bacteriostatic concentration	Lowest concentration able to inhibit microbial growth, without killing the test organism
Bactericidal concentration	Lowest concentration able to kill/inactivate the test microorganism

However, many papers propose direct contact between microorganism and antimicrobial agent; whereas, an alternative method is the use of essential oil in the vapor phase (Lopez et al., 2005; Tyagi et al., 2012). In the vapor diffusion assays a filter disk is impregnated with the antimicrobial agent and placed on the medium-free cover of each Petri dish, while microorganism target are inoculated on agar surface; the Petri dishes were then sealed using sterile adhesive tape.

Other methods are the micro-dilution approaches (dilution in broth or in agar, evaluation of microbial growth by plate counting or by indirect indices).

## Conflict of Interest Statement

The authors declare that the research was conducted in the absence of any commercial or financial relationships that could be construed as a potential conflict of interest.
